# Caffeine and physical performance in female intermittent sport athletes: a systematic review and meta-analysis considering menstrual cycle phase

**DOI:** 10.3389/fnut.2026.1817134

**Published:** 2026-05-22

**Authors:** Zhi Sen Tan, Stephen Francis Burns, Yun Xuan Lee, Alicia M. Goodwill

**Affiliations:** Physical Education and Sports Science Department, National Institute of Education, Nanyang Technological University, Singapore, Singapore

**Keywords:** caffeine, estrogen, female athletes, follicular, intermittent sports, luteal, menstrual cycle, physical performance

## Abstract

**Introduction:**

Female athletes remain underrepresented in caffeine-based performance research, and inconsistent menstrual cycle classification further limits generalization of the current evidence. This systematic review and meta-analysis examined the effects of caffeine on physical performance in female athletes participating in intermittent sports and explored whether these effects differ between the menstrual cycle phases, within the constraints of the available literature.

**Methods:**

A systematic search was conducted (PubMed, Scopus, Web of Science) from January 2000 to September 2025. Randomized controlled trials evaluating caffeine effects on physical performance in female athletes participating in intermittent-type sports and reporting menstrual cycle phase were included. Random-effects meta-analyses using standardized mean differences were conducted and subgroup analysis by menstrual cycle phase were pre-specified. Study quality was assessed using the Physiotherapy Evidence Database scale and Risk-of-Bias tool.

**Results:**

Nine studies (*n* = 118) were included, and six (*n* = 82) contributed to the meta-analysis. Caffeine was associated with improvements in agility (SMD = −0.62, 95% CI [−0.98 to −0.26], I^2^ = 0%) and vertical jump (SMD = 0.37, 95% CI [0.05 to 0.69], I^2^ = 0%) but not sprint performance (SMD = 0.03, 95% CI [−0.36 to 0.41], I^2^ = 15.2%) with low heterogeneity across outcomes. Within-condition sub-group analyses suggested caffeine-related improvements in agility within follicular-phase samples (SMD = −0.84, 95% CI [−1.34 to −0.33]), whereas effects in the luteal phase were less certain. However, between-group analyses showed no differences in caffeine effects between menstrual cycle phases (Q [1] = 1.41, *p* = 0.24), and these findings were based on a limited and methodologically constrained evidence base.

**Conclusion:**

Caffeine was associated with improvements in vertical jump and agility in females from intermittent sports. However, the small number of studies reporting menstrual cycle details, lack of appropriate experimental designs, and absence of rigorous menstrual cycle verification limit inference regarding phase-dependent effects. These findings highlight the need for well-designed studies to robustly test whether caffeine responses differ across menstrual cycle phases.

**Systematic review registration:**

https://www.crd.york.ac.uk/PROSPERO/view/CRD42025634451.

## 1 Introduction

Caffeine (1,3,7-trimethylxanthine) is an ergogenic aid, widely utilized by many athletes (1). This is supported by evidence demonstrating caffeine’s ergogenic effects on endurance exercise (2), short-term high intensity exercise (3), resistance exercise (4, 5), team-sport performance (6, 7), and ball sports (8, 9). Caffeine has been proposed to enhance sporting performance through several mechanisms. For example, increases in strength performance following caffeine has been associated with increased calcium ion (Ca^2+^) plasma concentrations (10) as well as the alteration of carbohydrate and fat oxidation rate (11). Most research centers on the premise that caffeine acts as a contender against adenosine at the receptors of the central nervous system (12). This dampens the effect of adenosine, boosting the release of neurotransmitters and dopamine (13, 14) and causing an upsurge in recruitment and activation of motor units (15).

Intermittent sports (e.g., basketball, soccer) are characterized by periodic, high-intensity efforts interspersed with lower intensity exercise. They require the execution of sport-specific skills and complex mental activities across a prolonged duration (16). These sports also have varied physical demands due to the need to perform multiple explosive actions such as sprinting, jumping, change of direction, acceleration, deceleration, and shuffling (17). Nutritional interventions which enhance these aspects of physical performance are potentially valuable. Several systematic reviews have shown that caffeine can improve several performance components of intermittent sports including vertical jump, sprint and agility performance in ball sports (8), team sports (18), soccer (19) and basketball (20). A recent systematic review and meta-analysis also found that caffeine increased the number of sprints, body impacts, accelerations, and decelerations during intermittent sport competitions (21). However, it is important to note that most of the subjects included in these prior reviews consisted of primarily male athletes (1,003 males out of 1,495 participants across five reviews, 67.1%). This issue was further highlighted in an audit by Smith and colleagues (22) on the representation of female athletes in sports science and medicine research, where more than half of the caffeine studies included in the audit (k = 858) consisted of male participants only. This underrepresentation of sport nutrition research in females may be in part due to sexual stereotypes, which have contributed to restricting female involvement in sports worldwide (23, 24). A recent qualitative critical discourse analysis revealed that social and economic factors such as low economic independence and insufficient media coverage on female sports are part of the many factors discouraging Iranian female participation in sports (24).

Besides social and gender stereotypes, another critical issue in female athlete physiology relates to methodological considerations surrounding the identification and influence of the menstrual cycle (25). Many experimental studies fail to control for ovarian hormonal fluctuations (estrogen and progesterone) across the menstrual cycle which can affect the quality of data collected and conclusions drawn (26). To date, one systematic review and meta-analysis has focused on the effects of caffeine on physical performance in female team-sport athletes (6) while another focused on identifying differences in ergogenicity of caffeine between sexes (19). It was found that caffeine improved countermovement jump (CMJ), squat jump (SJ) and single sprint performance, but did not improve agility and repeated sprint ability (RSA) in female team-sport athletes across 18 studies involving 240 female participants ranging in age from 18 to 26 years (6). Comparing between sexes, similar ergogenic effects of caffeine were found for aerobic performance and fatigue index (i.e., exercise-induced fatigue), while a greater effect was found in males for sprint performance and total power produced (19). However, both reviews included studies which did not report on the phase of the menstrual cycle when the testing was carried out, and only Mielgo-Ayuso et al. (19) briefly explained how the menstrual cycle influenced the absence of sex differences in knee extensor isometric contractions after consuming caffeine.

A recent review has extended this work by including menstrual cycle phase as a moderator (27). Findings indicated that the ergogenic effects of caffeine were significantly greater in the follicular phase. However, interpretation of these findings remains challenging, as many included studies did not clearly report the menstrual cycle phase during which testing took place. Moreover, previous reviews have primarily focused on specific performance outcomes in isolation and not on athletes participating in intermittent sports. This is important, considering intermittent sports involve repeated high-intensity efforts and rely on anaerobic performance capacities such as power, speed, and agility, which are key targets of caffeine’s ergogenic effects. Given that over half of elite female athletes subjectively report that their menstrual cycle affects their sporting performance (28, 29), the interaction between menstrual cycle phase and caffeine’s effects on performance related to intermittent sport warrants further investigation.

Estrogen and progesterone are the main hormones that fluctuate significantly across the menstrual cycle. These hormone concentrations can fluctuate more than 100% across 24 h, particularly in the transition from follicular to luteal phases (30). Specifically, in the early follicular phase, estrogen and progesterone levels are at their lowest, while during the mid-luteal phase, progesterone surges to a peak and estrogen reaches a second peak (31). Fluctuations in these hormones as a result of the menstrual cycle, may not only impact sports performance directly (29, 31), but also modulate the ergogenic effect of supplements such as caffeine. For example, estrogen has anabolic properties and can enhance glycogen absorption and spare glycogen stores (32). It is also suggested that estrogen can assist in reducing exercise-induced inflammation and hence, reduce muscle damage (32, 33). In contrast, progesterone has been proposed to oppose the beneficial effects of estrogen through catabolic pathways (34). While estrogen and progesterone have been suggested to affect the metabolism of caffeine and its ergogenic properties (35, 36), evidence regarding how these effects vary across the menstrual cycle remain mixed. For example, Lara and associates (36) suggested that caffeine experiences a more gradual breakdown into paraxanthine during the early follicular phase, which increases its ergogenic properties compared to the mid-luteal phase. Conversely, Sims and colleagues (37) challenged this notion by suggesting that slower elimination of caffeine is found in the luteal phase instead. These inconsistencies highlight an important gap regarding the extent to which the menstrual cycle phase modulates caffeine’s metabolic and ergogenic effects, particularly on physical performance related to intermittent sports. Therefore, following the “Population, Intervention, Comparator, Outcome” (PICO) framework (38), this systematic review and meta-analysis aimed to synthesize the effects of caffeine on physical performance in female athletes participating in intermittent sports, based on studies reporting menstrual cycle phase. A secondary aim was to explore whether performance responses vary between the follicular and luteal phase of the menstrual cycle.

## 2 Methods

### 2.1 Search strategy

This systematic review was performed in accordance with the Preferred Reporting Items for Systematic reviews and Meta-Analyses (PRISMA) guidelines and was prospectively registered in the International Prospective Register of Systematic Reviews (PROSPERO; CRD42025634451). The Assessing the Methodological Quality of Systematic Reviews version 2 (AMSTAR 2) checklist was also utilized as a guidance tool in the completion of this systematic review (39). Using three databases (PubMed, Scopus and Web of Science), a search was conducted from January 2000 to September 2025 using the combination of the following keywords: (‘caffeine’) AND (‘menstrual cycle’ OR ‘follicular phase’ OR ‘luteal phase’ OR ‘ovulation’ OR ‘estrogen’ OR ‘progesterone’ OR ‘pre-menopausal’) AND (‘intermittent sports performance’ OR ‘vertical jump’ OR ‘linear sprint’ OR ‘repeated sprint’ OR ‘agility’ OR ‘change-of-direction’; see Supplementary material A). The search was limited to studies published from 2000 onwards to reflect the period in which research on female participants, including reporting of menstrual cycle phase, became more consistently incorporated into exercise science studies. All article titles and abstracts were uploaded to Covidence (40) where duplicate articles were removed. Titles and abstracts were then screened for relevance, followed by full-text screening. The screening process was conducted manually by human reviewers, with no use of artificial intelligence or machine-learning-assisted tools. Title and abstract screening were performed by a single reviewer (ZST). Full-text articles were independently screened by two reviewers (ZST and YXL), with any disagreements to be resolved by a third reviewer (AMG); however, no conflicts arose and the involvement of AMG was not required.

### 2.2 Inclusion and exclusion criteria

The screened articles were included in the review for assessment based on these criteria: (1) studies that included female human participants, (2) studies that tested the effect(s) of caffeine on physical performances of intermittent sports, (3) included information on caffeine administration and dose, (4) reported the phase of participants’ menstrual cycle during testing, where available, (5) randomized placebo-controlled trial, including both parallel-group and crossover designs, with caffeine as the intervention, and (6) articles were available in English with full-texts and not abstracts only. No restrictions were placed on the method used to determine menstrual cycle phase due to the limited availability of studies. We defined female athletes as biologically female participants engaged in structured, recurrent sport. Intermittent sports were defined as sports characterized by high-intensity efforts interspersed lower-intensity efforts (e.g., basketball, soccer). Competitive level was not used as an inclusion criterion because the literature uses inconsistent terminology and because the available evidence base is limited.

**Figure 1:**
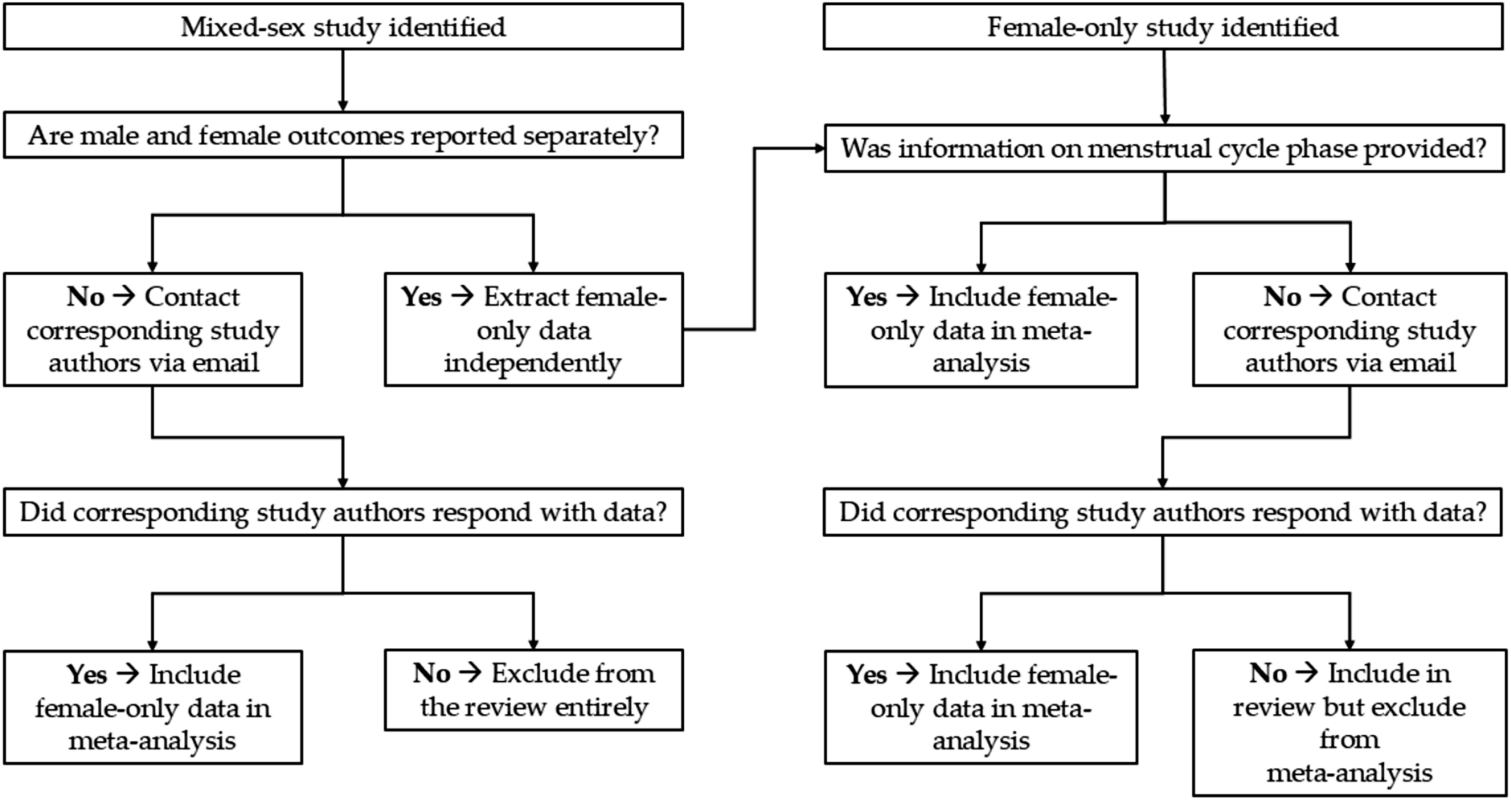
Schematic diagram of selection procedures for mixed-sex studies and female-only studies.

For mixed-sex studies (male and female) that did not provide female-only data and female-only studies that did not specify the menstrual cycle phase of their participants, corresponding authors were contacted via email to request for data of female athletes only and information on the menstrual cycle phase(s). When no response was received from the authors, female-only studies with data that were not distinguished by menstrual cycle phases were excluded from the meta-analysis while mixed-sex studies that did not provide data of female athletes only were excluded from this review entirely (Figure 1). Studies that did not measure tests reflecting physical performance crucial to intermittent sports (i.e., vertical jump performance, agility speeds, linear and repeated sprint performance) were also excluded from this review. This review was restricted to studies published in English only. Studies available exclusively in other languages, were excluded due to feasibility constraints in translation and data extraction. It is acknowledged that this language restriction may lead to omission of potentially relevant evidence. As such, the findings should be interpreted with the understanding that the evidence base may not fully represent all available literature on the topic.

### 2.3 Quality assessment and risk of bias

To assess the methodological quality of the studies, the Physiotherapy Evidence Database (PEDro) was used independently by two reviewers to examine the internal validity of randomized clinical trials. Studies were graded based on a total score of 10 points using these ranges: poor (0–3 points), fair (4–5 points), good (6–8 points), and excellent (9–10 points). Studies scoring below six were pre-specified (a-priori) as low methodological quality and would have been excluded (41) to ensure minimal standard of internal validity. No studies met this exclusion criteria. Notably, while the PEDro assesses general methodological quality, it does not capture domain-specific factors relevant to this review, such as the accuracy of menstrual cycle phase verification, which were considered separately.

To assess the risk of bias of each study, the Cochrane risk-of-bias (RoB 2) tool was used for randomized controlled trials following Cochrane Collaboration guidelines. The RoB 2 tool assesses the studies across six different domains: (1) randomization process, (2) period and carryover effects, (3) deviations from intended interventions, (4) missing outcome data, (5) measurement of outcome and, (6) selection of reported results. In each domain, information from the studies obtained was used to answer several signaling questions before an opinion was generated regarding the risk of bias. The opinions are categorized as: ‘low risk of bias’, ‘some concerns of bias’ and ‘high risk of bias’. Upon completing the assessment of the six domains, an overall risk-of-bias judgment was then obtained for each study by each of the two independent reviewers (ZST and YXL). The results of the PEDro and RoB 2 were then discussed by the reviewers to ensure that unanimity of assessment was achieved. When any conflicts arose, discussions were held with a third reviewer (AMG) until a consensus was reached.

The certainty of evidence for each performance outcome measure (vertical jump, sprints and agility) was evaluated using the Grading of Recommendations Assessment, Development, and Evaluation (GRADE) tool (42) across studies included in the meta-analysis (43–48). Each outcome measure was assessed across five domains: (1) risk of bias, (2) inconsistency, (3) indirectness, (4) imprecision and, (5) publication bias. The overall certainty grades were rated as either high, moderate, low or very low.

### 2.4 Data extraction

Data from each included study was extracted by one author (ZST) following an adapted version of the “Population, Intervention, Comparison, Outcome” (PICO) framework (Huang et al., 2006) and was cross-checked against the original studies to ensure accuracy. The following information was obtained: (1) author and year of study, (2) number and profile of participants, (3) menstrual cycle phase, (4) study design, (5) caffeine and placebo intervention administration details (dose, administration form and pre-exercise ingestion time), (6) performance outcome, (7) significance of caffeine vs. placebo on each outcome measure (vertical jump, agility, linear and repeated sprint). If multiple caffeine conditions were conducted within a study (e.g., more than one dose, timing of administration), the *p*-value corresponding to the overall main effect of caffeine was extracted for qualitative reporting. For the meta-analysis, mean and standard deviation data were extracted for caffeine and placebo according to menstrual cycle phase. When these were unavailable, corresponding authors were contacted to obtain the necessary information. Six of the nine authors responded, and these papers were included in the meta-analysis (43–48).

### 2.5 Statistical analyses

A random-effect, inverse-variance meta-analysis was performed to compare the overall pooled standardized mean differences ([SMDs], Hedges g) for each intermittent performance outcome measure (i.e., vertical jump height (cm), agility, and sprint speeds (s)) between caffeine and placebo conditions, where each study was given a weight. This method of analysis was selected because it was assumed that there would be variability in the intervention effect of caffeine. Exploratory within-condition subgroup meta-analyses were conducted with the available raw data for each condition, and the reported phase of the menstrual cycle (follicular and luteal phase) for each outcome measure. Differences between menstrual cycle phases were assessed the Cochran’s Q test. While the included studies reported information on the menstrual cycle phase during each experimental condition, no study conducted both caffeine and placebo conditions in both phases (i.e., no true within-subject design). Given that none of the included studies were explicitly designed to test for phase-specific interactions between caffeine and placebo, sub-group analyses are intended to guide hypotheses for future sports nutrition and menstrual cycle research, rather than establish causal interaction effects.

One study (45) administered two separate caffeine interventions (dose 3 and 6 mg/kg BM) for the same participants; therefore, this data was averaged using a weighted mean to obtain a single effect size for this study (49, 50). Similarly, if multiple performance measures were reported for the same category (e.g., CMJ with and without arm swing), these were averaged using a weighted mean to yield a single effect size for this study. The I^2^ statistic was utilized to test the heterogeneity of the studies, which expresses the variation between studies as a percentage of the total variance with the classification of low (25–49%), moderate (50–74%), and high (75–100%) heterogeneity (51). DerSimonian-Laird estimator was used as the default model by Comprehensive Meta-Analysis (CMA) for between-study variance of random-effects meta-analysis (50). Publication bias was assessed via funnel plots using Egger’s regression test (52), where significant asymmetry indicates presence of publication bias. No sensitivity analysis was conducted due to four reasons: (1) small sample size included, (2) no study was classified as high risk of bias, (3) similar study designs used with inadequate variability to test heterogeneity and, (4) multiple studies (4 of 9 studies) (43–46) originating from same research group. Having multiple studies from the same research group reduces the feasibility of excluding individual studies without disproportionately influencing the overall evidence base. All statistical analyses were completed using CMA (V4.0, Biostat, Englewood, United States) with statistical significance being set at alpha level of *p* < 0.05.

## 3 Results

### 3.1 Study characteristics

**Figure 2:**
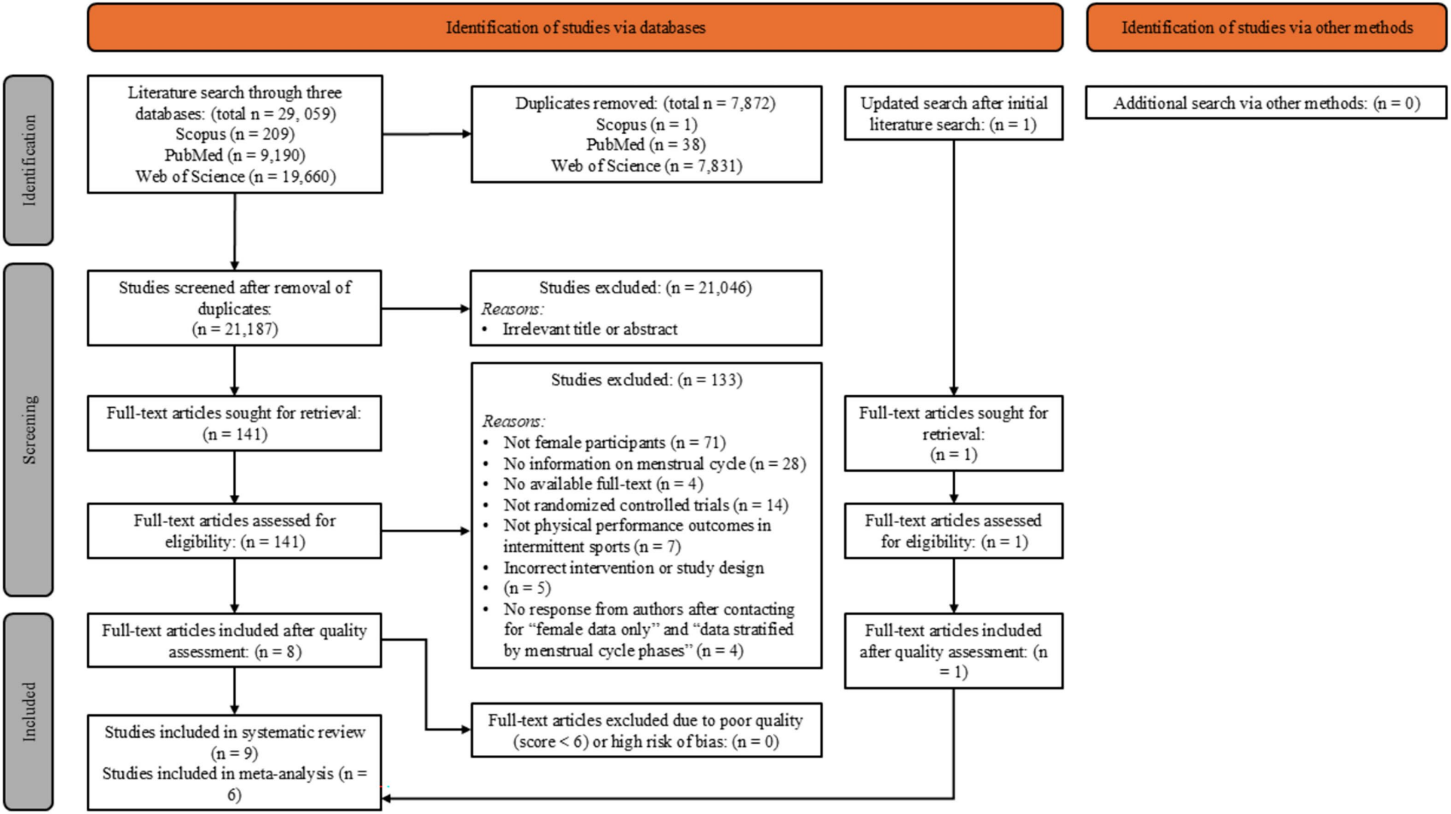
Search strategy and study selection process using PRISMA guidelines.

The literature search presented a total of 29,059 studies whereby 7,872 duplicate studies were removed. Of the remaining 21,187 studies, an additional 21,046 studies were removed due to irrelevancy of title and abstract. After the initial search was completed, an additional study was published, and the full text was screened for inclusion. Full-text review was performed by two independent reviewers (ZST and LYX) on 142 studies, and 133 studies were removed due to not being conducted on female participants (*n* = 71), absence of menstrual cycle information (*n* = 28), unavailability of full-text (*n* = 4), not randomized controlled trials (*n* = 14), not on physical performance outcomes in intermittent sports (*n* = 7), incorrect intervention or study design (*n* = 5), and no specification of female data only (*n* = 4). This resulted in nine studies (7, 9, 43–48, 53) fulfilling the inclusion criteria for the systematic review (Figure 2). As none of the studies included originally stratified their data by menstrual cycle sub-group, authors were contacted to provide this information and six of nine studies (43–48) responded with data that indicated the menstrual cycle phase their participants were tested in, for each of the caffeine and placebo conditions. These six studies were subsequently included in the meta-analysis. All nine included studies were randomized and performed within-group comparisons (caffeine vs. placebo), while eight specified that they were double-blind (7, 9, 43–48, 53). Only three studies mentioned that their study design was counterbalanced (44, 47, 48).

Results were obtained from 118 female athletes (ranging from 8 to 17 participants per study). Of these, 75 were handball athletes (~63.6%), 33 were volleyball athletes (~28.0%) and the remaining 10 were basketball athletes (~8.5%). Of the 75 handball athletes, 60 were youth athletes (80%) aged 15 to 19 years old. One article (53) also included youth volleyball athletes but did not specify the number of youth athletes out of eight participants.

### 3.2 Quality assessment and risk of bias

Assessing the internal validity of the studies using the PEDro scale, all nine studies were accepted and included in this review with scores ranging from 7 to 10 (see Supplementary material B). Six studies scored 9 to 10 and were classified as excellent while the remaining three studies scored 7 and 8 and were classified as good (41). Five of nine studies (7, 43, 44, 46, 53) did not meet the requirements for concealment of allocation while one study (43) did not elaborate on the blinding of therapists and assessors. Three studies (44–46) did not meet the requirements of obtaining outcome measures from more than 85% of participants.

For the RoB2 tool results, all nine studies were ascertained to be of low risk of bias in all domains. However, it is worth noting that three (44–46) were initially flagged as potential risks of bias under the missing outcome data domain (Domain 3) as 7 out of 20 participants (35%) (44), 5 out of 20 participants (25%) (45) and 7 out of 24 participants (~29.2%) (46) dropped out in each study. Further evaluation identified that the three studies did report on the reasons for dropout and that the participants were fully withdrawn - hence, missing data was not considered to be a source of bias. However, a potential research group bias was noted as four of nine studies (43–46) originated from the same laboratory. This raise concerns as the concentration of evidence from the same laboratory may limit the independence of findings. Overall, all nine papers were considered as low risk of bias using the RoB2 tool (Supplementary material C).

Using the GRADE tool to assess the certainty of evidence, all performance outcome measures (vertical jump, agility and sprint) of studies included in the meta-analysis (43–48) were rated as low (Supplementary material D). Evidence of each performance outcome measure was downgraded due to imprecision from the relatively small, pooled sample size, and indirectness due to variability in menstrual cycle phase identification.

### 3.3 Caffeine dose and administration

Across the nine studies reviewed, all except one (47) utilized caffeine doses provided in accordance with the participants’ body mass (BM) rather than an absolute dose. These doses ranged from 3 to 6 mg/kg BM, which is considered within the recommended range to elicit ergogenic benefits (54). Of these eight studies, three (7, 9, 48) utilized 3 mg/kg BM of caffeine, three (43, 44, 46) utilized a dose of 6 mg/kg BM of caffeine, one (53) used 5 mg/kg BM of caffeine, and one study administered both 3 and 6 mg/kg BM of caffeine in two different experimental sessions (45). In the study by Filip-Stachnik and colleagues (47), the authors utilized an absolute dose of 400 mg of caffeine.

With regards to administration time, all but one study (47) administered caffeine 60 min before the start of exercise. In terms of mode of administration, six administered via capsule (7, 43–46, 48), one mixed anhydrous caffeine powder into a maltodextrin beverage (53), and another dissolved caffeine-containing energy drink powder into water (9). The energy drink powder also contained 6.6 mg/kg BM of maltodextrin, but it was noted that the amount of exogenous energy from that was negligible (~2 kcal) (9). Lastly, Filip-Stachnik et al. (47) provided the caffeine dose through the form of chewing gum, and the gum was given 15 min before the start of exercise. The authors also highlighted that the use of an absolute dose was chosen as the provision of a relative dose was unfeasible via chewing gum.

### 3.4 Menstrual cycle phases

Only two of nine studies (45, 53) stated that their participants experienced eumenorrheic menstrual cycles. Siquier-Coll et al. (53) did not clearly define ‘eumenorrheic’ in their paper, while Bougrine et al. (45) reported regular menstrual cycles across the last 6 months (see Table 1). While all studies had reported on the phase of the menstrual cycle that testing took place in, eight did not report their descriptive data or stratify their analyses by menstrual cycle phase in the published paper. After contacting the authors, six studies (43–48) provided this data and were included in the meta-analysis.

**Table 1:** Menstrual cycle information of the nine included studies.

References	Eumenorrheic?	Menstrual cycle duration (days)	No. of participants in each phase per condition											
			Follicular phase						Luteal phase					
Muñoz et al. (7)	Not stated	Not stated	10						5					
Pérez-López et al. (9)	Not stated	Not stated	10						9					
Bougrine et al. (43)	Not stated	Not stated	CAF 0800 h	CAF 1800 h		PLA 0800 h	PLA 1800 h		CAF 0800 h	CAF 1800 h		PLA 0800 h	PLA 1800 h	
			12	11		11	12		3	4		4	3	
Bougrine et al. (44)	Not stated	Not stated	CAF & PLA 0800 h & 1800 h Pre-Ramadan			CAF & PLA 0800 h & 1800 h Post-Ramadan			CAF & PLA 0800 h & 1800 h Pre-Ramadan			CAF & PLA 0800 h & 1800 h Post-Ramadan		
			8			4			5			9		
Bougrine et al. (45)	Yes	27.8 ± 2	CAF 3 mg 0800 h	CAF 6 mg 0800 h	PLA 0800 h	CAF 3 mg 1800 h	CAF 6 mg 1800 h	PLA 1800 h	CAF 3 mg 0800 h	CAF 6 mg 0800 h	PLA 0800 h	CAF 3 mg 1800 h	CAF 6 mg 1800 h	PLA 1800 h
			8	9	8	4	4	8	7	6	7	11	11	7
Bougrine et al. (46)	Not stated	28 ± 1.9	CAF + MUS	CAF		PLA + MUS	PLA		CAF + MUS	CAF	PLA + MUS		PLA	
			12	7		12	12		5	10	5		5	
Stojanovic et al. (48)	Not stated	Not stated	0						10					
Filip-Stachnik et al. (47)	Not stated	Not stated	CAF & PLA											
			6						6					
Siquier-Coll et al. (53)	Yes – unclear definition	Not stated	CAF			PLA			CAF			PLA		
			3			4			5			6		

CAF, caffeine; PLA, placebo; MUS, music.

From the data provided of the six studies included in the meta-analysis, each condition (caffeine and placebo) was not compared in each menstrual cycle phase within the study. Hence, there was an unequal number of participants in each menstrual cycle phase of each condition for most of the included studies (Table 1). In addition, only one study (48) specifically stated that all their participants were in the luteal phase (*n* = 10).

Two methods of menstrual cycle identification were noted in six of the nine studies: (1) usage of mobile phone application to track (7, 43–46) and (2) calculation based on previously recorded menstrual cycle (53). The remaining three studies did not indicate how the menstrual cycle phase was identified (9, 47, 48). Using an adapted two item questionnaire to assess methodological quality of determining menstrual cycle phases (31), all nine studies scored 0 out of 2, indicating that none of them determined the menstrual cycle phase via hormonal concentrations from blood profiles or via urinary ovulation test kits (Supplementary material E).

### 3.5 Physical performance measures

All studies in this review measured vertical jump height. Six studies measured vertical jump height using the countermovement jump (CMJ) test (7, 9, 45, 46, 48, 53) with Stojanović and colleagues (48) measuring CMJ height with and without arm swing. Four studies measured vertical jump height using the squat jump (SJ) test (9, 43, 44, 48). In addition, Siquier-Coll et al. (53) used the 15 s repeated jump test (RJ-15) while Filip-Stachnik et al. (47) measured vertical jump height during sport-specific volleyball attack and block jumps. Of the six studies included in the meta-analysis, five provided vertical jump data for comparison between menstrual cycle phases (follicular vs. luteal phase) (43–47). Seven of nine studies (~77.8%) indicated that caffeine improved vertical jump performance (9, 43–47, 53). Specifically, as shown in Tables 2, 3, caffeine improved vertical jump performance for CMJ (9, 45, 46, 53), SJ (9, 43, 44), volleyball-specific attack and block jumps (47) as well as RJ-15 (53).

**Table 2:** Effect of caffeine on physical performances in intermittent sports (studies not included in meta-analysis).

References	Participant profile	Menstrual cycle information	Intervention	Administration mode and time (min)	Measures (Unit) & change	CAF	PLA	*p*
Muñoz et al. (7)	15 handball players [age: 22.6 ± 3.6 years, height; 170 ± 8 cm, BM: 69.5 ± 9.5 kg]; first division Spanish National League	10 follicular phase; 5 luteal phase	3 mg/kg BM of CAF or PLA; randomized, double-blind, crossover design	Capsule with 100 mL water; 60 min	Countermovement jump (cm): ↑	29.8 ± 5.5	28.5 ± 5.5	0.06
					Modified agility T test (s): -	-	-	0.669
					30 m sprint test (s): ↑	4.8 ± 0.3	4.9 ± 0.2	0.042*
Pérez-López et al. (9)	13 volleyball players [age: 25.2 ± 4.8 years; height: 174 ± 9 cm; BM: 64.4 ± 7.6 kg]; Second division Spanish National League	4 follicular phase; 9 luteal phase	3 mg/kg BM of CAF or PLA; randomized, double-blind design	Energy drink powder dissolved in 250 mL water; 60 min	Countermovement jump (cm): ↑	33.1 ± 4.5	32.0 ± 4.6	0.018*
					Squat jump (cm): ↑	29.4 ± 3.6	28.1 ± 3.2	0.028*
					Agility T test (s): ↑	10.9 ± 0.3	11.1 ± 0.5	0.036*
Siquier-Coll et al. (53)	8 volleyball players [age: 17 to 25 years; height: 163 ± 8 cm; BM: 66.7 ± 4.7 kg]; Spanish Women’s Superleague 2	3 follicular, 5 luteal phase for CAF condition 4 follicular, 4 luteal phase for PLA condition	5 mg/kg BM of CAF or PLA; randomized, double-blind, crossover design	Anhydrous powder mixed with maltodextrin beverage; 60 min	MD-4			
					Countermovement jump (cm): ↑	34.2 ± 5.6	30.7 ± 4.4	0.02*
					Repeated jump 15″ (cm): ↑	28.3 ± 2.8	26.2 ± 2.6	0.04*
					Change of direction 505 test (s): -	4.32 ± 0.19	4.31 ± 0.23	-
					Yo-Yo IR1 (m): -	-	-	-
					MD-3			
					Countermovement jump (cm): ↑	37.2 ± 4.7	34.7 ± 6.0	0.02*
					Repeated jump 15″ (cm): ↑	30.3 ± 2.4	28.5 ± 4.5	0.04*
					Change of direction 505 test (s): -	4.17 ± 0.19	4.17 ± 0.19	-
					Yo-Yo IR1 (m): -	-	-	-
					MD-1			
					Countermovement jump (cm): ↑	35.5 ± 6.1	33.9 ± 7.0	0.02*
					Repeated jump 15″ (cm): ↑	30.3 ± 3.4	27.8 ± 3.0	0.04*
					Change of direction 505 test (s): -	4.13 ± 0.14	4.15 ± 0.11	-
					Yo-Yo IR1 (m): -	-	-	-

Data are reported as mean ± SD. CAF, caffeine; PLA, placebo; BM, body mass; - no change; ↑ increased; * significant change (*p* < 0.05).

**Table 3:** Effect of caffeine on physical performances in intermittent sports (studies included in meta-analysis).

References	Participant profile	Menstrual cycle information	Intervention	Administration mode and time (min)	Measures (Unit) & change	CAF	PLA		*p*
Bougrine et al. (43)	15 handball players [age: 16.3 ± 0.8 years; height: 166 ± 5 cm; BM: 58.7 ± 9.1 kg]; First division Tunisian National League	Follicular phase, luteal phase or both	6 mg/kg BM of CAF or PLA; randomized, cross-over design	Capsule with 100 mL water; 60 min	Morning				
					Squat jump (cm): ↑	29.99 ± 1.69	28.78 ± 1.67		<0.001*
					Illinois agility test (s): ↑	19.05 ± 0.71	19.37 ± 0.86		<0.001*
					5 m shuttle run test best (m): ↑	689.3 ± 37.0	673.2 ± 35.2		< 0.001*
					5 m shuttle run test total (m): ↑	124.27 ± 6.67	117.27 ± 6.42		<0.001*
					Evening				
					Squat jump (cm): ↑	30.89 ± 1.84	30.59 ± 1.73		<0.001*
					Illinois agility test (s): ↑	18.96 ± 0.73	19.0 ± 0.72		<0.001*
					5 m shuttle run test best (m): ↑	696.5 ± 37.7	694.7 ± 38.1		<0.001*
					5 m shuttle run test total (m): ↑	128.2 ± 6.56	127.0 ± 6.47		<0.001*
Bougrine et al. (44)	13 handball players [age: 16.6 ± 0.5 years; height: 170 ± 1 cm; BM: 59.3 ± 9.1 kg]; First level of Tunisian National League	Follicular phase, luteal phase or both	6 mg/kg BM of CAF or PLA; randomized, cross-over, counterbalanced, double-blind design	Capsule with 150 mL water; 60 min	Morning Pre-Ramadan				
					Squat jump (cm): ↑	28.2 ± 1.6	26.9 ± 1.3		<0.001*
					Illinois agility test (s): ↑	18.7 ± 0.5	19.1 ± 0.6		<0.01*
					5 m shuttle run test best (m): ↑	683.2 ± 35.9	667.8 ± 34.6		<0.001*
					5 m shuttle run test total (m): ↑	123.1 ± 6.3	116.1 ± 6.0		<0.001*
					Evening Pre-Ramadan				
					Squat jump (cm): -	29.2 ± 1.7	28.9 ± 1.6		>0.05
					Illinois agility test (s): -	18.5 ± 0.5	18.6 ± 0.5		>0.05
					5 m shuttle run test best (m): -	690.5 ± 36.8	688.7 ± 37.3		>0.05
					5 m shuttle run test total (m): ↑	127.0 ± 6.2	125.8 ± 6.2		<0.05*
					Morning Post-Ramadan				
					Squat jump (cm): ↑	27.8 ± 1.7	26.7 ± 1.3		<0.001*
					Illinois agility test (s): ↑	18.7 ± 0.5	19.07 ± 0.6		<0.001*
					5 m shuttle run test best (m): ↑	682.2 ± 36.6	666.8 ± 35.1		<0.001*
					5 m shuttle run test total (m): ↑	121.6 ± 6.0	114.9 ± 6.2		<0.001*
					Evening Post-Ramadan				
					Squat jump (cm): ↑	28.7 ± 1.7	28.1 ± 1.7		<0.05*
					Illinois agility test (s): ↑	18.7 ± 0.5	18.9 ± 0.5		<0.01*
					5 m shuttle run test best (m): ↑	686.0 ± 37.0	677.3 ± 37.4		<0.001*
					5 m shuttle run test total (m): ↑	124.6 ± 5.95	121.0 ± 6.0		<0.001*
Bougrine et al. (46)	17 handball players [age: 16.8 ± 0.4 years; height: 164 ± 10 cm; BM: 59.4 ± 6 kg]	Follicular phase, luteal phase or both	6 mg/kg of CAF or PLA; randomized, cross-over, double-blind design	Capsule with 100 mL water; 60 min	With Music				
					Countermovement jump (cm): ↑	23.41 ± 1.67	22.71 ± 1.67		< 0.001* < 0.001* < 0.001* < 0.001*
					Modified agility test (s): ↑	8.08 ± 0.26	8.34 ± 0.17		
					Peak RSA (s): ↑	7.06 ± 0.29	7.13 ± 0.30		
					Mean RSA (s): ↑	7.26 ± 0.29	7.43 ± 0.26		
					Without Music				
					Countermovement jump (cm): ↑	23.32 ± 1.69	22.36 ± 1.73		<0.001* < 0.001* < 0.001* < 0.001*
					Modified agility test (s): ↑	8.14 ± 0.27	8.46 ± 0.17		
					Peak RSA (s): ↑	7.08 ± 0.29	7.25 ± 0.29		
					Mean RSA (s): ↑	7.30 ± 0.26	7.51 ± 0.24		
Filip-Stachnik et al. (47)	12 volleyball players [age: 20 ± 2 years; height: 178 ± 6 cm; BM: 69.1 ± 2.3 kg]; Second division Polish National League	6 follicular phase; 6 luteal phase	400 mg of CAF or PLA; randomized, cross-over, counterbalanced, double-blind design	Chewing gum; 15 min	Pre-game attack jump (cm): ↑	47.2 ± 7.3	46.0 ± 7.9		0.024*
					Post-game attack jump (cm): ↑	47.5 ± 7.5	46.3 ± 8.3		
					Pre-game block jump (cm): -	33.0 ± 4.5	32.6 ± 5.7		0.724
					Post-game block jump (cm): -	34.7 ± 6.2	34.8 ± 6.4		
Stojanović et al. (48)	10 basketball players [age: 20.2 ± 3.9 years; height: 175 ± 6 cm; BM: 69.2 ± 6.3 kg]; First division Serbian National League	All luteal phase	3 mg/kg BM of CAF or PLA; randomized, cross-over counterbalanced, double-blind design	Capsule with 250 mL water; 60 min	Countermovement jump w arm swing (cm): -	35.1 ± 5.1	33.9 ± 3.9		0.15
					Countermovement jump w/o arm swing (cm): -	29.2 ± 4.4	27.9 ± 4.2		0.10
					Squat jump (cm): ↑	27.2 ± 4.4	26.0 ± 3.2		0.08
					Lane agility drill (s): -	13.0 ± 0.9	13.2 ± 0.9		0.12
					5 m sprint (s): -	1.18 ± 0.11	1.24 ± 0.15		0.13
					10 m sprint (s): ↑	2.01 ± 0.13	2.11 ± 0.18		0.05
					20 m sprint (s): ↑	3.49 ± 0.23	3.59 ± 0.25		0.04*
					Suicide run (s): -	31.8 ± 1.62	32.2 ± 1.74		0.28
References	Participant profile	Menstrual cycle information	Intervention	Administration mode and time (min)	Measures (Unit) & change	3 mg/kg BM CAF	6 mg/kg BM CAF	PLA	*p^α^*
Bougrine et al. (45)	15 handball players [age: 18.3 ± 0.5 years; height: 165 ± 6 cm; BM: 60.2 ± 5.7 kg]	Follicular phase, luteal phase or both	3 mg/kg BM or 6 mg/kg BM of CAF or PLA; randomized, cross-over, double-blind design	Capsule with 100 mL water; 60 min	Morning				
					Countermovement jump (cm): ↑	22.8 ± 1.8	23.1 ± 1.8	22.2 ± 1.7	<0.001*
					Modified agility T test (s): ↑	8.5 ± 0.4	8.2 ± 0.3	8.6 ± 0.4	<0.001*
					Mean RSA test (s): ↑	7.4 ± 0.3	7.2 ± 0.2	7.5 ± 0.2	<0.001*
					Peak RSA test (s): -	7.1 ± 0.3	7.0 ± 0.3	7.2 ± 0.3	0.29
					Evening				
					Countermovement jump (cm): ↑	22.9 ± 1.9	23.1 ± 1.9	22.2 ± 1.7	<0.001*
					Modified agility T test (s): ↑	8.0 ± 0.4	8.0 ± 0.4	8.6 ± 0.4	<0.001*
					Mean RSA test (s): ↑	7.3 ± 0.2	7.2 ± 0.2	7.5 ± 0.2	<0.001*
					Peak RSA test (s): -	7.0 ± 0.3	7.0 ± 0.2	7.2 ± 0.3	0.29

Data are reported as mean ± SD. CAF, caffeine; PLA, placebo; BM, body mass; RSA, repeated sprint ability; - no change; ↑ increased; * significant change (*p* < 0.05). p^α^ denotes the significance of the main effect of dosage in ANOVA.

Seven of nine studies measured the effects of caffeine on linear and repeated sprint performance (7, 43–46, 48, 53) but only four of the seven provided sprints data for comparison across menstrual cycle phases (43–46). Sprint performance was measured using 30 m linear sprints (7), repeated sprint ability (RSA) (45, 46), 5 m shuttle run test (5mSRT) (43, 44), Yo-Yo Intermittent Recovery Test 1 (YYIRT) (53), 5 m, 10 m and 20 m linear sprints as well as suicide runs on a basketball court (48). Six of seven studies (~85.7%) showed that 3 mg/kg and 6 mg/kg BM of caffeine improved most measures of sprint performance (7, 43–46, 48) (Tables 2, 3). The study by Siquier-Coll et al. (53) did not provide any data regarding the outcome of the YYIRT following caffeine consumption.

All but one study (47) analyzed the effect of caffeine on agility or change-of-direction in female athletes with four of the eight studies providing data for menstrual cycle phase comparison (43–46). The Lane Agility Drill (LAD) (48), Illinois Agility Test (IAT) (43, 44), modified agility T-test (7, 45, 46), 505 test (53) and agility T-test (9) were used to measure the change-of-direction speed. One of eight studies (12.5%) showed that 3 mg/kg BM of caffeine improved change-of-direction speed (9) while four studies (50%) indicated that 6 mg/kg BM of caffeine improved change-of-direction speed (43–46) (Tables 2, 3).

### 3.6 Meta-analyses

Six of nine studies provided data of participant outcome measures in each menstrual cycle phase and were included in the meta-analysis (43–48). The pooled statistics of the meta-analysis revealed that a positive effect favoring caffeine was found for agility performance (SMD = −0.622, 95% CI [−0.981 to −0.264], *p* = 0.001) and vertical jump (SMD = 0.366, 95% CI [0.046 to 0.686], *p* = 0.025) but not for sprint (SMD = 0.025, 95% CI [−0.363 to 0.413], *p* = 0.898) performance. As agility performance is measured in time, a negative mean difference indicates that agility test time was faster following the consumption of caffeine.

**Figure 3:**
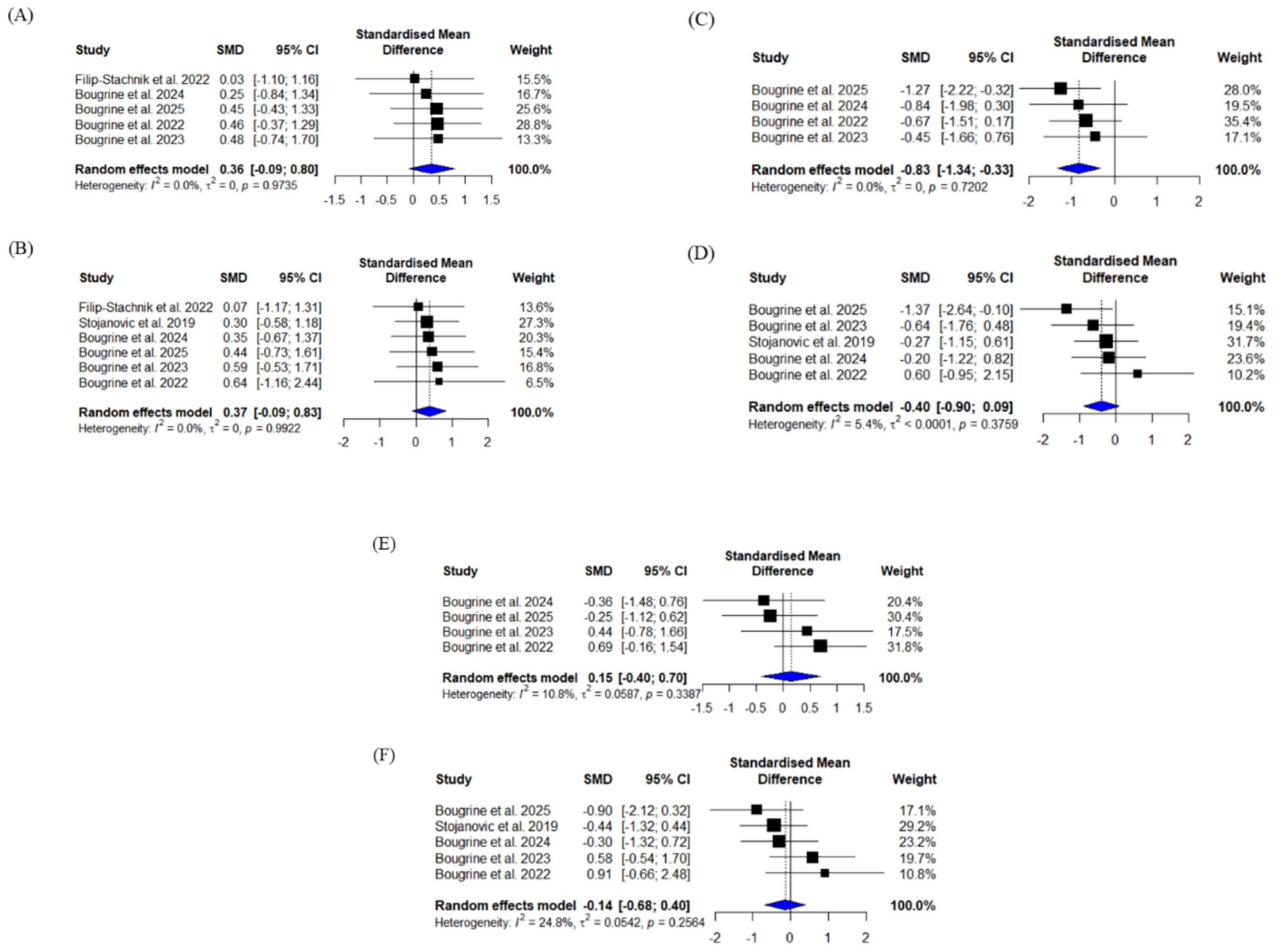
Forest plots of studies using caffeine to improve physical performance in female intermittent sport athletes. **(A)** Vertical jump performance in follicular phase, **(B)** vertical jump performance in luteal phase, **(C)** agility performance in follicular phase, **(D)** agility performance in luteal phase, **(E)** sprint performance in follicular phase, and **(F)** sprint performance in luteal phase. For vertical jump, positive scores favor caffeine, while for agility and sprint performance, negative scores favor caffeine.

Exploratory, within-group subgroup analyses suggested caffeine improved agility performance in the follicular phase trials (Figure 3C) whereas the effect in the luteal phase was uncertain (Figure 3D). However, between-group comparisons showed no statistically significant differences between the menstrual cycle phases [Q (1) = 1.406, *p* = 0.236]. Exploratory subgroup analyses on vertical jump (Figures 3A,B) and sprint performance (Figures 3E,F) suggest no effect within either menstrual cycle phase.

**Figure 4:**
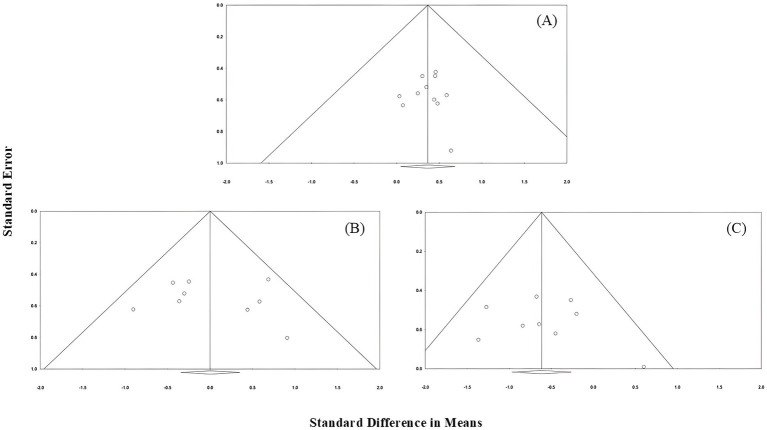
Funnel plot illustrates the level of publication bias of all studies included in the meta-analysis. **(A)** Vertical jump performance, **(B)** sprint performance, and **(C)** agility performance.

The pooled statistics also revealed that heterogeneity between the studies was low for all outcome measures [vertical jump: Q (1) = 0.002, *p* = 0.968; agility: Q (1) = 1.406, *p* = 0.236; sprints: Q (1) = 0.485, *p* = 0.486]. The funnel plot of all included studies revealed a symmetrical distribution and Egger’s regression test revealed no indication of any publication bias for vertical jump [*t* (9) = 0.012, *p* = 0.991], agility [*t* (7) = 0.579, *p* = 0.581] and sprints [*t* (7) = 0.368, *p* = 0.724; Figure 4].

## 4 Discussion

This review examines caffeine effects on physical performance in female intermittent sport athletes while considering menstrual cycle phase. Although previous reviews have examined caffeine use in female team-sport athletes (6) and explored the menstrual cycle phase as a potential moderator (27), none have synthesized this evidence using meta-analysis focused on studies reporting menstrual cycle phase in intermittent sport populations. The present systematic review included nine studies (7, 9, 43–48, 53) and the meta-analysis included a subset of six studies (43–48), reflecting the limited evidence base. Within this context, three observations emerged: (1) caffeine was associated with improvements in agility and vertical jump performance, (2) no clear effect was observed for sprint performance, and (3) sub-group analyses suggested no differences in caffeine-related performance outcomes between menstrual cycle phases. Importantly, as the included studies were not explicitly designed to test caffeine-menstrual cycle interactions, and phase was not manipulated within participants, these findings should be interpreted as exploratory and hypothesis generating. Together, these findings highlight a critical gap in the literature, as current study designs lack the methodological rigor required to robustly evaluate menstrual cycle–dependent responses to caffeine in female athletes. Thus, the primary contribution of this review lies not only in synthesizing available evidence, but in demonstrating that current study designs are insufficient to robustly evaluate menstrual cycle–dependent responses to caffeine. This also reinforces that our current understanding of how female athletes respond to supplements, such as caffeine, often falls short of the stronger evidence base that exists for their male counterparts.

This meta-analysis observed improvements in vertical jump performance from caffeine, which aligns with a recent meta-analysis on caffeine and female team-sports (6). In the present analysis, vertical jump was treated as a pooled outcome rather than separated by jump type (e.g., CMJ vs. SJ), and subgroup analyses suggested no clear moderation by menstrual cycle phase. Given the limited number of studies and lack of phase-controlled designs, these findings should be interpreted with caution. To advance understanding in this area, future studies should systematically control and report menstrual cycle phase when assessing neuromuscular performance outcomes.

For sprint performance, the present meta-analysis observed no effect of caffeine compared with placebo, similar to the findings from Gomez-Bruton and colleagues (6). In contrast, evidence regarding agility performance remains mixed. A previous meta-analysis reported that caffeine improved agility speeds across eight studies (18) while Gomez-Bruton et al. (6) found no effect of caffeine on agility in female team-sports performance across six studies. The current meta-analysis (*n* = 5) indicated that caffeine was associated with agility performance, consistent with the findings of Salinero and colleagues (18). Notably, there was minimal overlap in included studies across these reviews, with only one study (48) shared with Gomez-Bruton et al. (6), and none with Salinero et al. (18), highlighting differences in the evidence base.

While the performance findings provide some evidence for ergogenic effects of caffeine, interpretation of menstrual cycle-effects remains limited. Previous work considered menstrual cycle phase as a potential moderator of caffeine-induced changes in performance outcomes (27), although this review did not restrict inclusion to studies reporting menstrual cycle phase. In the present analysis, within-phase analyses suggested improvements in follicular-phase trials, whereas findings in the luteal-phase were less consistent; however, no significant differences were found between phases. These findings should be interpreted cautiously, as they are based on a small number of studies (k = 4 to 6), with a substantial proportion of participants drawn from a narrow subset of athletes (predominantly youth handball players), potentially increasing the disproportionate influence of individual studies, and sensitivity analyses were not feasible. Furthermore, variability in menstrual cycle classification methods and the use of different agility tests reduces comparability across studies. Greater consistency in both menstrual cycle phase determination and performance assessment is needed to improve the quality and generalisability of future research.

Several methodological factors further limit interpretation of phase-specific effects. For example, menstrual cycle phase was typically defined using a binary approach (follicular vs. luteal), despite evidence that the cycle can be segmented into multiple sub-phases with distinct hormonal profiles (26). This may introduce variability within phases and limit the precision of phase-based comparisons. In addition, much of the existing literature examining menstrual cycle effects has focused on aerobic or endurance-based outcomes (55–57), whereas the performance measures in the present review were primarily anaerobic or neuromuscular in nature. None of the included studies verified menstrual cycle phase using hormonal measures (e.g., blood profiling or ovulation testing), relying instead on methods such as calendar-based tracking. This approach assumes regular ovulation timing, which may not always be accurate (31), and further reduces the reliability of phase classification. Consequently, proposed mechanisms involving estrogen, progesterone, and caffeine metabolism (e.g., CYP1A2 activity) (58) could not be evaluated within the current evidence base. Greater consistency and rigor in menstrual cycle verification, including the use of gold-standard tracking methods and more precise phase definitions, are needed to improve the quality and interpretability of future research examining caffeine responses across the menstrual cycle.

Beyond these methodological considerations, it is increasingly recognized that female athletes remain largely underrepresented in sports nutrition and performance research as compared to their male counterparts (24), particularly in studies examining the effects of caffeine on performance. This reflects a broader structural landscape and barriers to female participation in sport, including sociocultural and institutional factors (23, 24). Therefore, the significance of this review is further accentuated, as it responds to this evidence gap in sports nutrition and performance research in females and highlights menstrual cycle considerations that are seldom addressed in the literature.

### 4.1 Limitations

While this review provides a synthesis of the available evidence, its primary contribution lies in highlighting key limitations of the current literature, and several important methodological constraints should be acknowledged. Firstly, the included studies did not employ a true 2 × 2 within-subject design, whereby both caffeine and placebo were administered across both follicular and luteal phases within the same participants. Instead, testing was conducted according to the menstrual cycle phase at the time of assessment, without controlling supplement exposure across phases. As such, menstrual cycle phase functioned as a between-subject characteristic rather than a within-subject factor, potentially increasing variability. Secondly, although studies were rated as low risk of bias using PEDro and RoB2 tools, four were conducted in the same laboratory (43–46), which could limit the independence of pooled estimates and reduces the generalisability of findings. Additionally, the use of only mobile applications or calendar tracking to identify menstrual cycle limits confidence in phase classification and interpretation of follicular-luteal phase comparisons. Finally, inconsistent reporting of factors such as contraceptive use, habitual caffeine intake, and training volume may have further influenced outcomes.

## 5 Conclusion

This meta-analysis indicates that caffeine is associated with improvements in agility and vertical jump performance in female athletes participating in intermittent sports. While analyses within menstrual cycle phases suggested improvements in agility in follicular-phase trials, no differences were observed between phases. Given the absence of appropriately designed interaction studies and the small, methodologically constrained evidence base, these findings should be interpreted as exploratory. Overall, the current literature remains insufficient to robustly evaluate menstrual cycle–dependent responses to caffeine.

## Data Availability

Publicly available datasets were analyzed in this study. This data can be found at: NIE data repository, doi: https://doi.org/10.25340/R4/7HSLJO.
