# Supplementary figures and images for: Caffeine and physical performance in female intermittent sport athletes: a systematic review and meta-analysis considering menstrual cycle phase
Source: Front Nutr. 2026 May 22;13:1817134. doi: 10.3389/fnut.2026.1817134 (PMC13236873; doi:10.3389/fnut.2026.1817134)

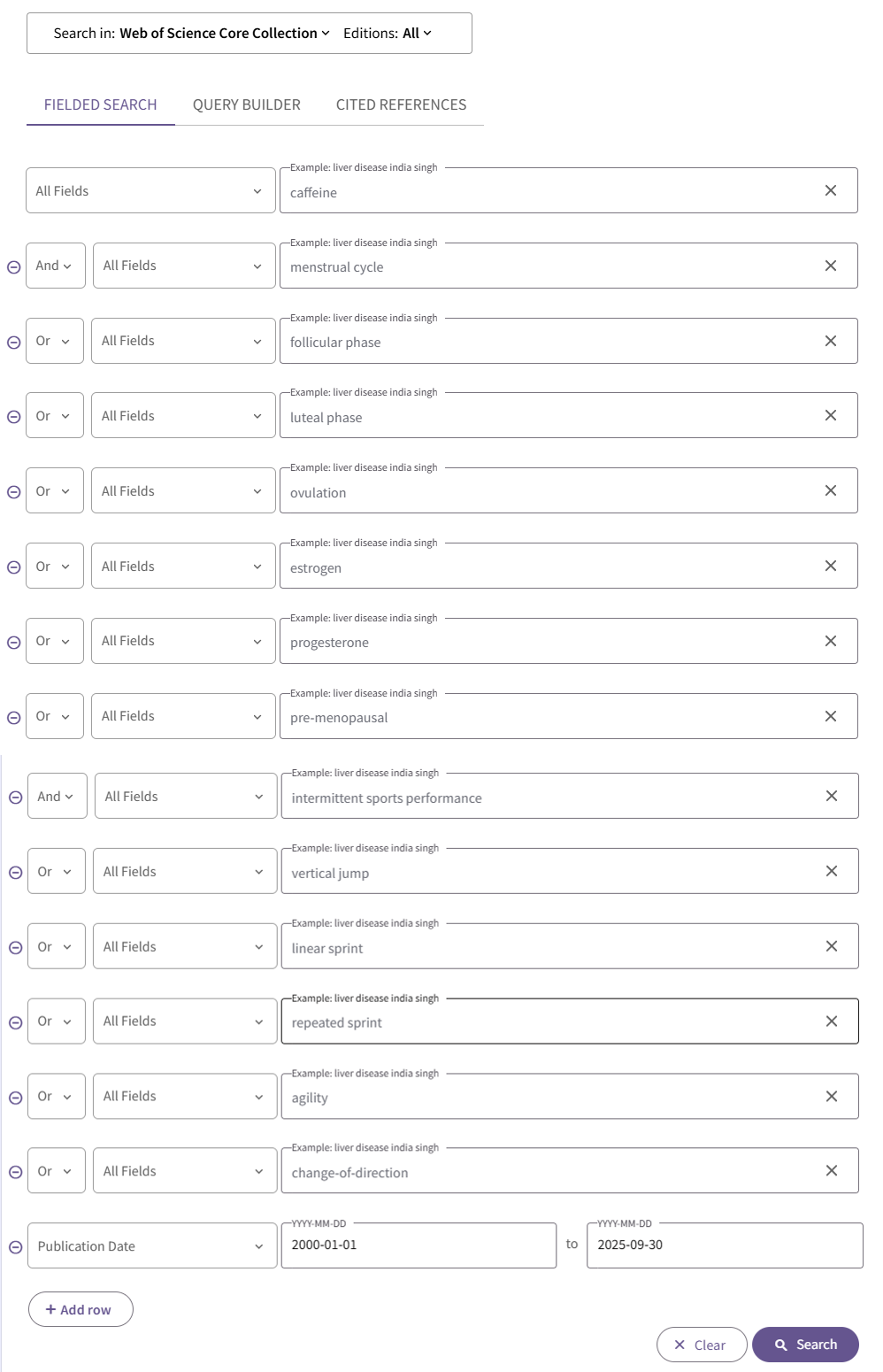

Supplement: Supplementary file 1 [file Image_1.png]

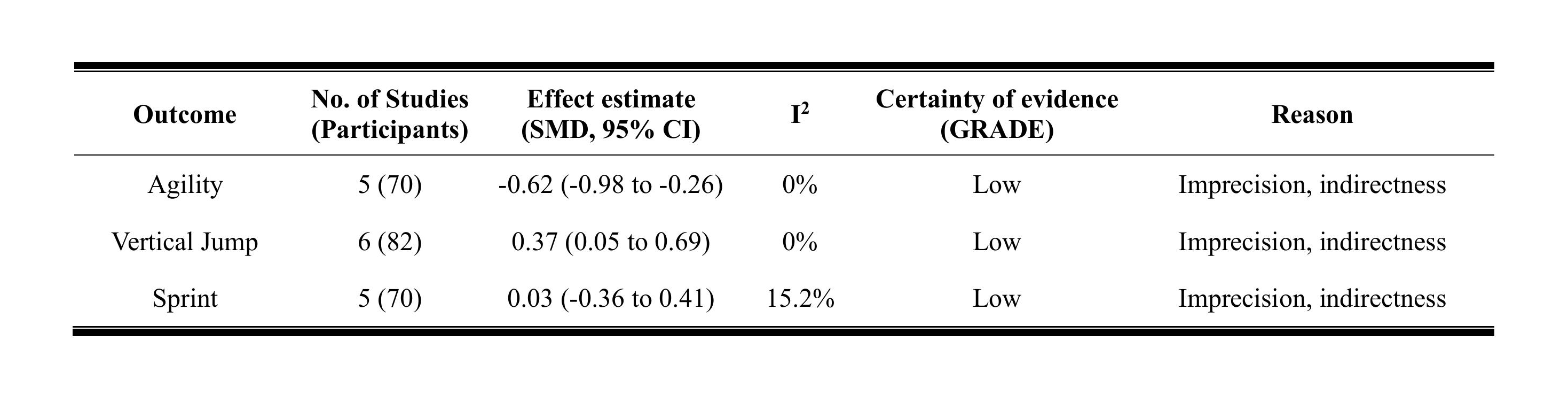

Supplement: Supplementary file 2 [file Image_2.tiff]

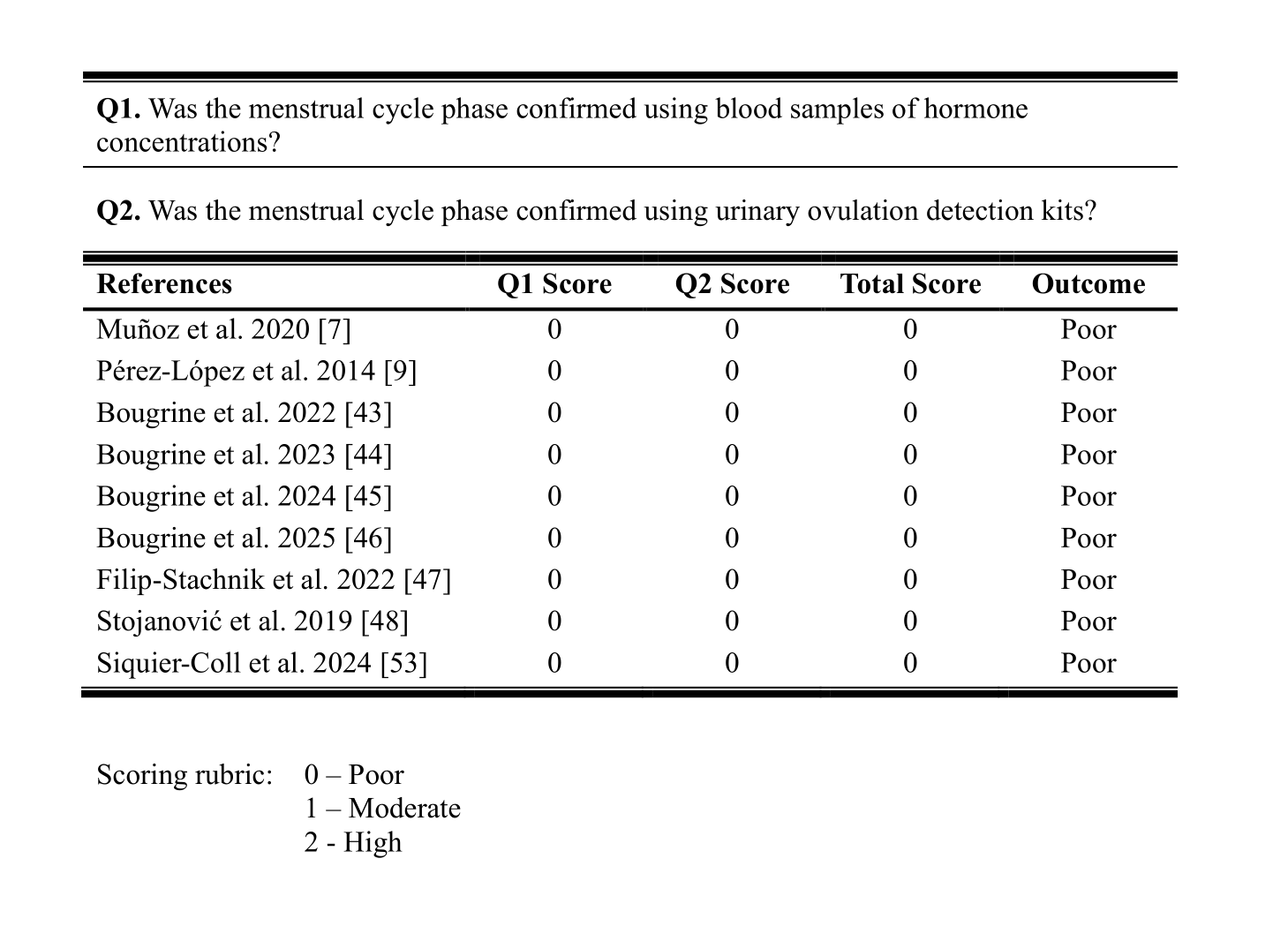

Supplement: Supplementary file 3 [file Image_3.tiff]

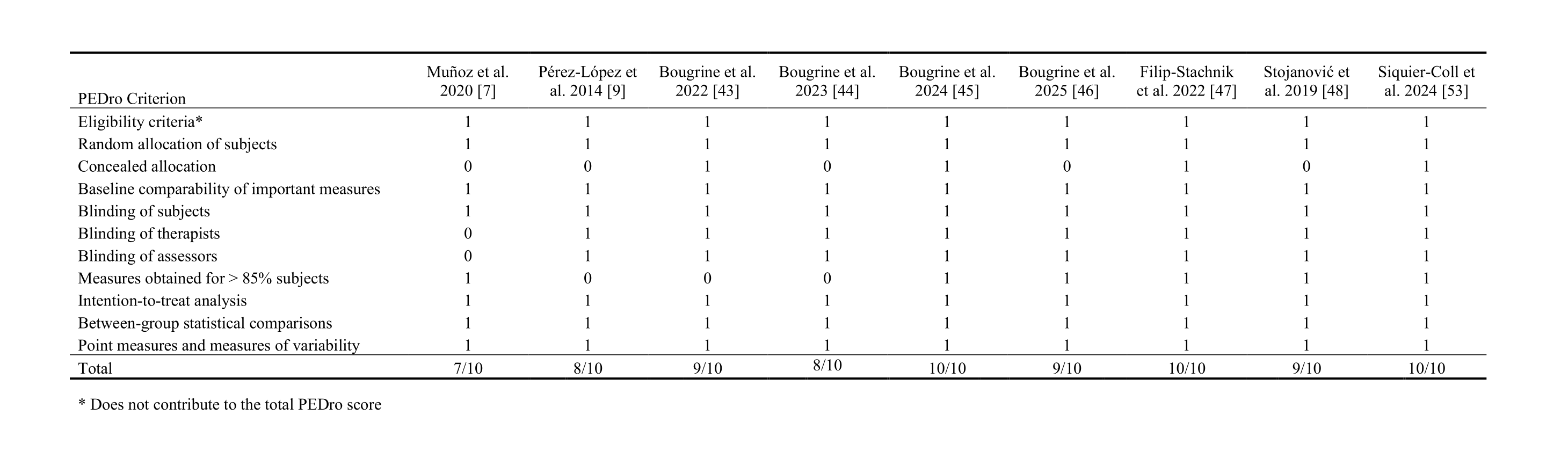

Supplement: Supplementary file 4 [file Image_4.tiff]

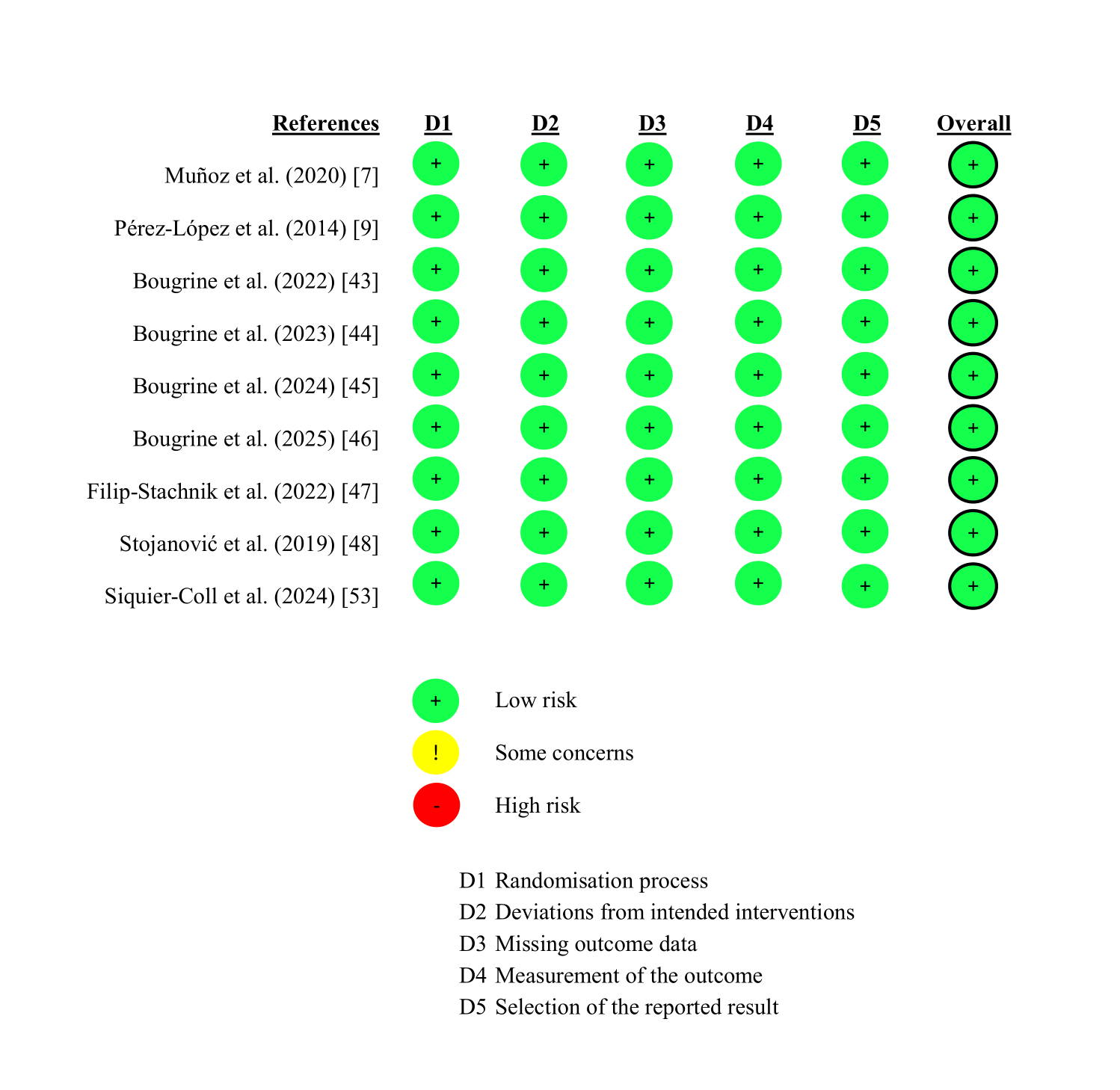

Supplement: Supplementary file 5 [file Image_5.tiff]
